# Non‐Linear Association of Relationship Between Serum Vitamin E and Eczema in US Adults

**DOI:** 10.1002/fsn3.71265

**Published:** 2025-11-24

**Authors:** Tianhang Yu, Peiyu Wang, Suhua Wu, Xueyun Cheng, Linfeng Li

**Affiliations:** ^1^ Department of Dermatology Beijing Friendship Hospital, Capital Medical University Beijing China

**Keywords:** eczema, NHANES, nonlinearity, oxidative stress, serum vitamin E

## Abstract

Eczema is an inflammatory skin disorder that influences 10%–20% of the global population. Previous research has shown that vitamin E supplementation may alleviate eczema symptoms, but the relationship between serum vitamin E and eczema incidence remains unclear. This study was a cross‐sectional analysis utilizing data from the 2005–2006 US National Health and Nutrition Examination Survey (NHANES). We included data from adult participants who were surveyed for eczema and tested for vitamin E. Multivariate logistic regression models were employed to investigate the relationship between serum vitamin E levels and eczema. The generalized additive model (GAM) was employed to investigate the nonlinear relationship, while a two‐piecewise linear regression model was utilized to identify the inflection point. The analysis included 4433 adults. The prevalence of eczema was 7.65%. The mean serum vitamin E concentration was 29.18 μmol/L (95% CI: 28.54–29.83). After adjusting for participants' demographics, way of life, stress, and clinical comorbidity variables, a non‐linear association was detected between serum vitamin E and eczema incidence, which had two inflection points for serum vitamin E at 27.4 and 49.5 μmol/L. Serum vitamin E levels < 27.4 μmol/L were positively associated with the eczema incidence (OR = 1.064, 95% CI: 1.024–1.105; *p* = 0.001), whereas levels between 27.4 and 49.5 μmol/L showed an inverse association (OR = 0.960, 95% CI: 0.934–0.987; *p* = 0.004). Vitamin E demonstrates a nonlinear association with eczema in US adults. Serum vitamin E levels < 27.4 μmol/L showed a positive association with eczema, whereas levels between 27.4 and 49.5 μmol/L demonstrated an inverse association.

## Graphical abstract

This study found a significant nonlinear association between serum vitamin E levels and eczema incidence. Serum vitamin E levels < 27.4 μmol/L were positively associated with eczema incidence, whereas levels between 27.4 and 49.5 μmol/L showed an inverse association.

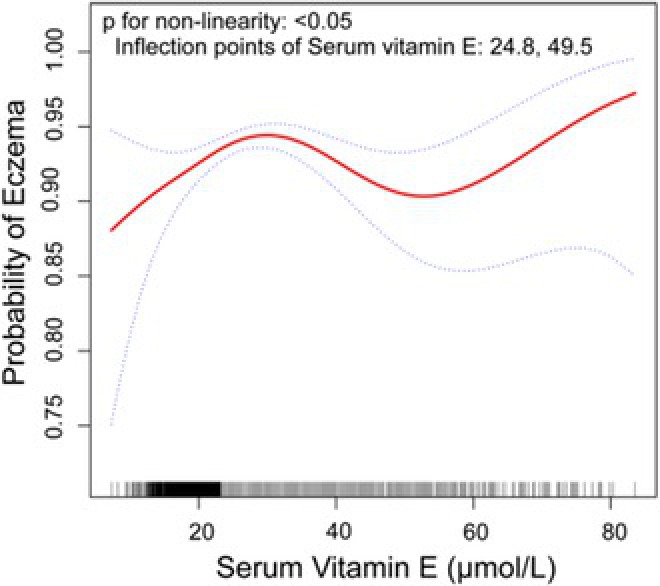

## 1 Introduction

Eczema is the most common chronic pruritic dermatological condition, impacting around 15%–20% of youngsters and 10% of older people globally (Stefanovic et al. 2020; Teo et al. 2021; Wei et al. 2019). It is a complex, multifactorial condition involving oxidative stress and inflammation in its pathogenesis. Recently, the role of oxidative stress (OS) in eczema has garnered widespread attention (Teo et al. 2021; Xu and Li 2024). At high concentrations, reactive oxidants induce epidermal keratinocyte damage through lipid peroxidation, thereby disrupting skin barrier function (Teo et al. 2021). Additionally, OS promotes the production of pro‐inflammatory cytokines, activates naive T cells, and triggers dermal cell infiltration, consequently exacerbating eczema lesions (Xu and Li 2024).

Vitamin E is a lipophilic antioxidant with antioxidant and anti‐inflammatory bioactivities and helps upregulate gene expression of keratinocyte differentiation markers, which play a key role in skin health (Teo et al. 2021). A‐tocopherol is the main component and the most biologically active form of vitamin E in humans (Ağagündüz et al. 2023; Choi et al. 2020; Reboul et al. 2006), and it can be better absorbed and retained by human cells. Numerous studies have used α‐tocopherol to explore vitamin E levels (Teo et al. 2021). α‐Tocopherol can neutralize lipid peroxidation free radicals (Gehin et al. 2006), protect cell membranes and regulate Th1/Th2 balance (Zhang 2023).

Much research have investigated the association between vitamin E and eczema, but this link remains unclear (Huang et al. 2024). Several randomized controlled trials have shown that vitamin E supplementation can improve the clinical symptoms of eczema and allergic reactions (Faghihi et al. 2015; Javanbakht et al. 2010, 2011; Tsoureli‐Nikita et al. 2002). Notably, while research has found an inverse association between serum α‐tocopherol concentrations and eczema prevalence among Japanese students (Okuda et al. 2010), a European multicenter cohort study did not observe the association between serum vitamin E and eczema in children (Nwaru et al. 2014). These conflicting results suggest that the relationship may not be a simple linear one.

We hypothesized a non‐linear relationship and a threshold saturation effect between serum vitamin E and eczema. In this study, we used data from the 2005–2006 National Health and Nutrition Examination Survey (NHANES), a nationally representative cross‐sectional survey of non‐institutionalized US residents. This study sought to investigate the possible association between serum α‐tocopherol levels and eczema prevalence among US adults.

## 2 Methods

### 2.1 Study Design and Population

NHANES is an ongoing, population‐based, cross‐sectional survey administered by the National Center for Health Statistics (NCHS) of the Centers for Disease Control and Prevention (CDC) (Wang et al. 2024). NHANES utilizes a sophisticated, multistage probability sampling methodology to obtain a nationally representative sample of the noninstitutionalized population in the United States. The NCHS Research Ethics Review Board authorized the survey methodology, and informed permission was acquired from all participants (Zhu et al. 2021).

**FIGURE 1:**
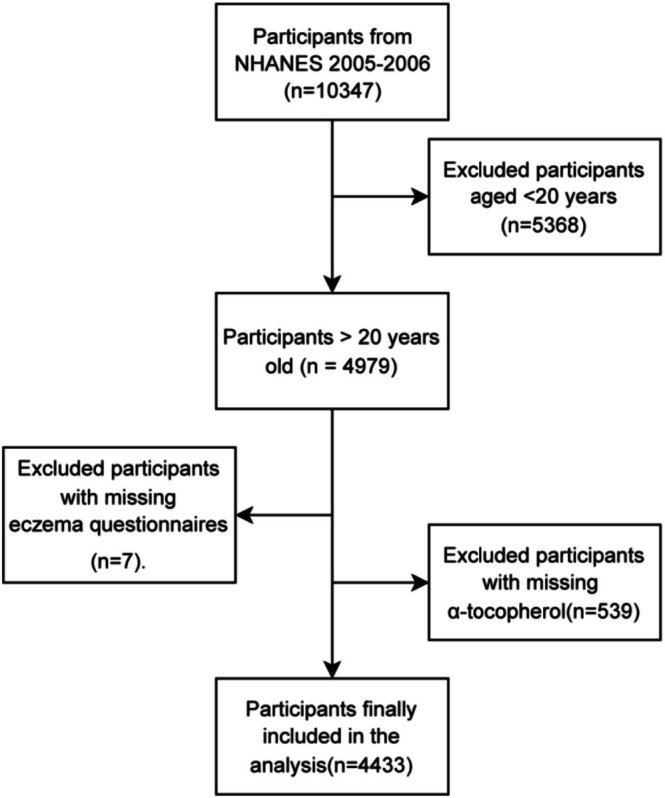
Participant selection flow chart.

This cross‐sectional research collected data from 4979 participants over the age of 20 in the 2005–2006 NHANES cycle. Although the 1999–2000, 2001–2002, and 2003–2004 NHANES cycles contained self‐reported eczema data, and serum α‐tocopherol measurements were also available in the 1999–2000 and 2003–2004 cycles, the 2005–2006 cycle was the only one that assessed doctor‐diagnosed eczema data and was therefore used in the present analysis (Wei et al. 2019). We incorporated a total of 4433 individuals into our statistical analysis after excluding participants with missing eczema status (*n* = 7) or serum vitamin E measurements (*n* = 539). Figure 1 displays a flowchart for the inclusion and exclusion of participants.

### 2.2 Exposure and Outcome Definitions

Exposure variables are serum vitamin E (α‐tocopherol). Serum α‐tocopherol levels were collected from participants (Deng et al. 2023; Teo et al. 2021). Serum α‐tocopherol is quantified by high‐performance liquid chromatography with photodiode array sensing. Serum specimens are processed, stored, and sent to the National Center for Environmental Health for examination (Zhang et al. 2017). The NHANES quality assurance and inspection processes comply with the mandates of the 1988 Clinical Laboratory Improvement Amendments. Comprehensive protocols for the sampling, processing, transportation, testing, and quality control of vitamin E and other covariates can be found at http://cdc.gov/nchs/nhanes. Serum vitamin E levels were defined according to clinical thresholds as deficient (< 12 μmol/L), adequate (12–30 μmol/L), or optimal (> 30 μmol/L) (Lebold et al. 2012; Péter et al. 2015).

Whether the participant has ever had eczema is considered an outcome variable. Eczema was defined through the question, “Has a doctor or other health professional ever told you that you have eczema?” If the answer is yes, they are classified as having eczema. If the answer is no, they are classified as not having eczema (Zhang et al. 2023).

### 2.3 Covariates

According to previous literature (Wei et al. 2019; Wei and Ji 2020), we collected demographic, way of life, stress, and clinical comorbidity data as covariates for analysis.

Demographic variables included age, gender, season (summer, winter), race (Mexican American, Other Hispanic, Non‐Hispanic Black, Non‐Hispanic White, and Other Race), poverty income ratio (PIR), and education level (less than 9th grade, 9–11th grade, high school graduate, some college or associate's degree, and college graduate or higher).

Way of life variables included body mass index (BMI, kg/m^2^), smoking (Yes, No), and drinking alcohol (Yes, No). BMI is calculated as weight divided by the square of height. Smokers were classified as those having consumed a minimum of 100 cigarettes in life. Drinkers were defined as persons who consumed a minimum of 12 alcoholic drinks during 1 year (Ran et al. 2024).

Stress related variables included sleep, categorized into three groups (< 6 h, 6–7 h, and > 7 h per night), and depression (none, mild, moderate, and severe). Depression was identified through the Patient Health Questionnaire (PHQ). The PHQ is a valid and dependable self‐reported evaluation based on the nine depressive symptoms outlined in the Diagnostic and Statistical Manual of Mental Disorders, the fourth edition (DSM‐IV) (Arroll et al. 2010). Total PHQ measures were stratified into four clinical categories: none (scores 0–4), slight (scores 5–9), moderate (scores 10–14), and severe (scores 15–27).

Clinical comorbidity variables included hay fever and asthma. Hay fever was defined through the question “Has a doctor or other health professional ever told you that you have hay fever?” If the answer is yes, they are classified as having hay fever. If the answer is no, they are classified as not having hay fever. Asthma was defined through the inquiry “Has a physician or other health professional previously told you that you have asthma?” If the answer is yes, they are classified as having asthma. If the answer is no, they are classified as not having asthma. We also adjusted serum immunoglobin E (IgE) as a biomarker of inflammation in allergic diseases.

### 2.4 Statistical Analysis

According to guidelines provided by the US CDC, we considered the intricate survey design of NHANES using stratification (SDMVSTRA) and clustering (SDMVPSU) variables with 2‐year examination weights (WTMEC2YR) for 2005–2006. Continuous data are expressed as weighted averages with 95% confidence intervals (CIs), while categorical data are presented as weighted proportions (95% CIs). *p*‐values for continuous variables were calculated using survey‐weighted linear regression, and for categorical variables using the design‐adjusted Rao–Scott chi‐squared test (Chen et al. 2025; Ciardullo et al. 2022).

A generalized linear model was employed to evaluate the relationship between serum vitamin E and eczema. The paper listed both non‐adjusted models and multivariate adjusted models. In accordance with the recommendations of the STROBE declaration (von Elm et al. 2007), we presented the analytical findings of the unadjusted, minimally adjusted, and fully adjusted analyses concurrently. We employed generalized additive models (GAM) and curve fitting to investigate the nonlinear association between serum vitamin E and eczema. If a nonlinear relationship was observed, a recursive algorithm was used to calculate inflection points in the relationship between serum vitamin E and eczema, and analyzed threshold effects. Nonlinearity was assessed by comparing the one‐line linear model and the two‐piecewise linear model using a design‐adjusted Wald F test appropriate for the NHANES complex survey design.

We used R version 4.2.0 (http://www.R‐project.org, The R Foundation) and Empower Stats 4.0 (http://www.empowerstats.com, X&Y Solutions Inc. Boston, MA, USA) for statistical analysis. Two‐sided *p* < 0.05 was considered statistically significant.

### 2.5 Sensitivity Analysis

We did further sensitivity analyses to ensure the stability of the statistical analyses. To address missing data in our covariates, we conducted multiple imputation (MI) with five replications through the chained equation method in the R MI process (Graham 2009; Haukoos and Newgard 2007). We conducted comparative analyses of population characteristics between the original dataset and the five MI‐generated datasets. We made fitted curves to these 6 data to determine whether the data generated by MI were consistent with the original data core results.

## 3 Result

The analysis included 4433 U.S. adults from the NHANES database with complete data for the exposure variable (vitamin E) and outcome variable (eczema). The study comprised 48.15% males (95% CI: 46.83% to 49.47%) and 51.85% females (95% CI: 50.53% to 53.17%). The mean age was 46.72 years (95% CI: 45.29 to 48.14). The mean serum vitamin E concentration was 29.18 μmol/L (95% CI: 28.54–29.83). The prevalence of ever‐reported eczema among U.S. adults was 7.65%.

### 3.1 Participants Characteristics at Baseline

Table 1 presents the baseline characteristics of participants categorized by clinical vitamin E threshold. Participants in the optimal vitamin E group (> 30 μmol/L) were more likely to be older, female, non‐Hispanic White, possess a college education or higher, have a higher PIR, have lower total IgE levels, be non‐smokers, report no depressive symptoms, sleep 6–7 h per night, and have no history of hay fever or asthma. There were no statistically significant differences across vitamin E categories with respect to BMI, alcohol consumption, sampled season, or eczema prevalence.

**TABLE 1:** The baseline characteristics of study participants.

Serum vitamin E, μmol/L	< 12 (deficient)	12–30 (adequate)	> 30 (optimal)	*p*
Participants, *n*	23	2831	1579	
Age, years	42.01 (34.61, 49.42)	42.48 (41.41, 43.55)	54.24 (52.48, 55.99)	< 0.0001
Gender				
Male	55.65 (22.76, 84.24)	49.53 (47.50, 51.56)	45.62 (43.23, 48.04)	0.0477
Female	44.35 (15.76, 77.24)	50.47 (48.44, 52.50)	54.38 (51.96, 56.77)	
BMI, kg/m^2^	29.70 (24.16, 35.24)	28.63 (28.09, 29.16)	28.51 (28.04, 28.99)	0.8626
Total IgE, KU/L	188.73 (61.43, 316.03)	142.47 (129.01, 155.94)	108.98 (87.67, 130.28)	0.0177
Race				
Mexican American	3.75 (0.35, 30.44)	8.82 (6.83, 11.33)	6.60 (4.76, 9.10)	< 0.0001
Other Hispanic	5.03 (0.20, 57.71)	3.67 (2.11, 6.31)	2.74 (1.56, 4.76)	
Non‐Hispanic White	71.94 (41.16, 90.38)	68.25 (61.64, 74.20)	79.93 (74.23, 84.63)	
Non‐Hispanic Black	19.28 (5.97, 47.33)	13.90 (9.65, 19.61)	5.91 (3.83, 9.01)	
Other Race	0.00 (0.00, 0.00)	5.35 (4.10, 6.95)	4.82 (3.30, 6.99)	
Education level				
Less than 9th grade	5.72 (0.66, 35.59)	6.69 (5.19, 8.58)	5.97 (4.48, 7.90)	< 0.0001
9–11th grade	24.85 (5.39, 65.73)	13.06 (10.31, 16.42)	7.49 (5.88, 9.49)	
High school graduate	44.31 (9.42, 85.89)	24.49 (22.89, 26.17)	24.95 (20.84, 29.56)	
Some college or associate's degree	16.69 (2.35, 62.50)	32.50 (29.53, 35.61)	29.63 (26.00, 33.54)	
College graduate or higher	8.43 (0.36, 70.37)	23.26 (18.65, 28.61)	31.96 (27.05, 37.31)	
PIR	1.98 (1.37, 2.59)	2.96 (2.80, 3.11)	3.43 (3.30, 3.57)	< 0.0001
Drinking alcohol status				
Yes	79.02 (33.24, 96.61)	74.11 (69.24, 78.45)	74.10 (70.00, 77.82)	0.9123
No	20.98 (3.39, 66.76)	25.89 (21.55, 30.76)	25.90 (22.18, 30.00)	
Smoking status				
Yes	65.39 (29.34, 89.58)	50.65 (47.49, 53.80)	45.66 (41.86, 49.51)	0.0083
No	34.61 (10.42, 70.66)	49.35 (46.20, 52.51)	54.34 (50.49, 58.14)	
Season				
Summer	49.93 (15.86, 84.07)	60.40 (46.15, 73.07)	55.90 (41.57, 69.31)	0.0524
Winter	50.07 (15.93, 84.14)	39.60 (26.93, 53.85)	44.10 (30.69, 58.43)	
Depression				
None	58.20 (18.77, 89.34)	79.20 (76.71, 81.48)	82.58 (79.24, 85.49)	0.0090
Mild	27.19 (5.29, 71.40)	14.54 (12.86, 16.41)	13.61 (11.47, 16.08)	
Moderate	7.21 (0.30, 66.80)	4.20 (3.38, 5.21)	2.59 (1.78, 3.76)	
Severe	7.40 (0.37, 63.17)	2.06 (1.33, 3.16)	1.21 (0.73, 2.01)	
Sleep				
< 6 h	24.40 (4.81, 67.30)	14.68 (13.29, 16.19)	10.57 (8.83, 12.60)	0.0108
6–7 h	48.17 (8.70, 90.07)	52.65 (50.16, 55.12)	52.79 (49.25, 56.30)	
> 7 h	27.44 (4.08, 77.08)	32.67 (29.77, 35.71)	36.64 (32.86, 40.59)	
Eczema				
Yes	10.17 (1.07, 54.31)	7.92 (6.51, 9.61)	7.14 (5.00, 10.10)	0.6944
No	89.83 (45.69, 98.93)	92.08 (90.39, 93.49)	92.86 (89.90, 95.00)	
Asthma				
Yes	11.36 (2.37, 40.42)	15.62 (13.87, 17.54)	11.16 (9.56, 12.98)	< 0.0001
No	88.64 (59.58, 97.63)	84.38 (82.46, 86.13)	88.84 (87.02, 90.44)	
Hay fever				
Yes	8.36 (0.35, 70.10)	11.79 (10.14, 13.67)	15.16 (13.45, 17.04)	0.0084
No	91.64 (29.90, 99.65)	88.21 (86.33, 89.86)	84.84 (82.96, 86.55)	

*Note:* Results in table: For continuous variables: survey‐weighted mean (95% CI), *p*‐value was by survey‐weighted linear regression. For categorical variables: survey‐weighted percentage (95% CI), *p*‐value was by design‐adjusted Rao–Scott chi‐squared test. Serum vitamin E, α‐tocopherol.

Abbreviations: BMI, body mass index; IgE, immunoglobin E; PIR, poverty income ratio.

Additionally, baseline characteristics of participants categorized by vitamin E quartiles are shown in Table S1. The proportions of non‐Hispanic White individuals, higher PIR, and history of hay fever increased progressively from the lowest levels (Q1) to the highest (Q4) quartile of serum vitamin E.

### 3.2 Univariate Analysis

Table 2 shows the results of the univariate analysis. Univariate analysis showed that female participants were negatively associated with eczema compared to males. In terms of race/ethnicity, compared with Mexican Americans, Other Hispanic, Non‐Hispanic Black, Non‐Hispanic White, and Other Race groups were all negatively associated with eczema. Regarding education level, compared with people with less than a 9th‐grade education, those who were high school graduates, had some college or an associate's degree, or were college graduates or higher were less likely to have eczema. Compared with summer sampling, winter sampling was positively associated with eczema. In terms of stress and comorbidities, compared with the absence of depressive symptoms, moderate and severe depressive symptoms were negatively associated with eczema. Furthermore, compared with participants with asthma, those without asthma were positively associated with eczema, and similarly, those without hay fever were positively associated with eczema.

**TABLE 2:** The results of univariate analysis.

	No. of participants	OR (95% CI)	*p*
Age, years	4433	1.00 (1.00, 1.01)	0.285
Gender			
Male	2138	Ref	
Female	2295	0.65 (0.53, 0.81)	0.001
BMI, kg/m^2^	4368	1.00 (0.98, 1.02)	0.821
IgE, KU/L	4426	1.00 (1.00, 1.00)	0.093
Race			
Mexican American	902	Ref	
Other Hispanic	136	0.40 (0.17, 0.94)	0.060
Non‐Hispanic White	2240	0.18 (0.12, 0.28)	< 0.001
Non‐Hispanic Black	983	0.24 (0.16, 0.36)	< 0.001
Other Race	172	0.29 (0.15, 0.54)	0.002
Education level			
Less than 9th grade	545	Ref	
9–11th grade	682	0.38 (0.16, 0.92)	0.055
High school graduate	1046	0.37 (0.19, 0.74)	0.017
Some college or associate's degree	1268	0.32 (0.16, 0.66)	0.010
College graduate or higher	887	0.30 (0.13, 0.69)	0.016
PIR	4234	0.92 (0.84, 1.01)	0.117
Drinking alcohol status			
Yes	2837	Ref	
No	1283	1.03 (0.68, 1.55)	0.907
Smoking status			
Yes	2102	Ref	
No	2328	1.17 (0.89, 1.54)	0.276
Season at sampling			
Summer	2414	Ref	
Winter	2019	1.85 (1.26, 2.71)	0.007
Depression			
None	3227	Ref	
Mild	581	0.77 (0.46, 1.30)	0.344
Moderate	170	0.54 (0.34, 0.86)	0.023
Severe	83	0.20 (0.10, 0.40)	0.001
Sleep			
< 6 h	667	Ref	
6–7 h	2175	0.94 (0.69, 1.28)	0.706
> 7 h	1581	0.88 (0.64, 1.21)	0.457
Serum vitamin E, μmol/L			
> 30 (optimal)	1579	Ref	
12–30 (adequate)	2831	0.89 (0.65, 1.24)	0.509
< 12 (deficient)	23	0.68 (0.18, 2.50)	0.571
Asthma			
Yes	571	Ref	
No	3856	1.98 (1.41, 2.78)	0.001
Hay fever			
Yes	460	Ref	
No	3961	2.41 (1.61, 3.62)	0.001

Abbreviation: Ref, reference.

### 3.3 The Association Between Serum Vitamin E Levels and Eczema

Table 3 illustrates the association between serum vitamin E levels and eczema evaluated using three logistic regression models. In the unadjusted model (without covariates adjusted), Model I (adjusted for sex and age) and Model II (adjusted for age, gender, season, education level, PIR, BMI, smoking status, drinking alcohol status, depression, sleep, asthma, hay fever, and IgE), continuous serum vitamin E concentrations showed no association with eczema risk. When serum vitamin E was categorized according to clinical thresholds, no significant associations with eczema were observed for the adequate (12–30 μmol/L) and deficient (< 12 μmol/L) groups compared with the optimal group (> 30 μmol/L) across all three models.

**TABLE 3:** Relationship between serum vitamin E and eczema in different models.

Serum vitamin E, μmol/L	Crude model (OR, 95% CI)	*p*	Model I (OR, 95% CI)	*p*	Model II (OR, 95% CI)	*p*
Serum vitamin E	1.003 (0.993, 1.015)	0.534	1.002 (0.990, 1.014)	0.743	1.000 (0.988, 1.013)	0.958
Serum vitamin E clinical threshold						
> 30 (optimal)	Ref		Ref		Ref	
12–30 (adequate)	0.89 (0.65, 1.24)	0.509	1.029 (0.790, 1.340)	0.831	1.083 (0.812, 1.444)	0.589
< 12 (deficient)	0.68 (0.18, 2.50)	0.571	0.456 (0.133, 1.571)	0.214	0.440 (0.117, 1.649)	0.223

*Note:* crude model: we did not adjust other covariates. Model I: we adjusted age, gender. Model II: we adjusted demographic variables (age, gender, season, education level, PRI), way of life variables (smoking status, drinking alcohol status, BMI), stress variables (depression, sleep), and clinical comorbidity variables (asthma, hay fever, IgE).

In Table S2, serum vitamin E concentrations were also analyzed by quartiles (Q1–Q4), using the lowest quartile (Q1: < 21.20 μmol/L) as the reference, Participants in the third quartile (Q3: 26.47–33.67 μmol/L) consistently showed a significantly increased risk of eczema across all models.

### 3.4 The Analyses of Non‐Linear Relationship

**FIGURE 2:**
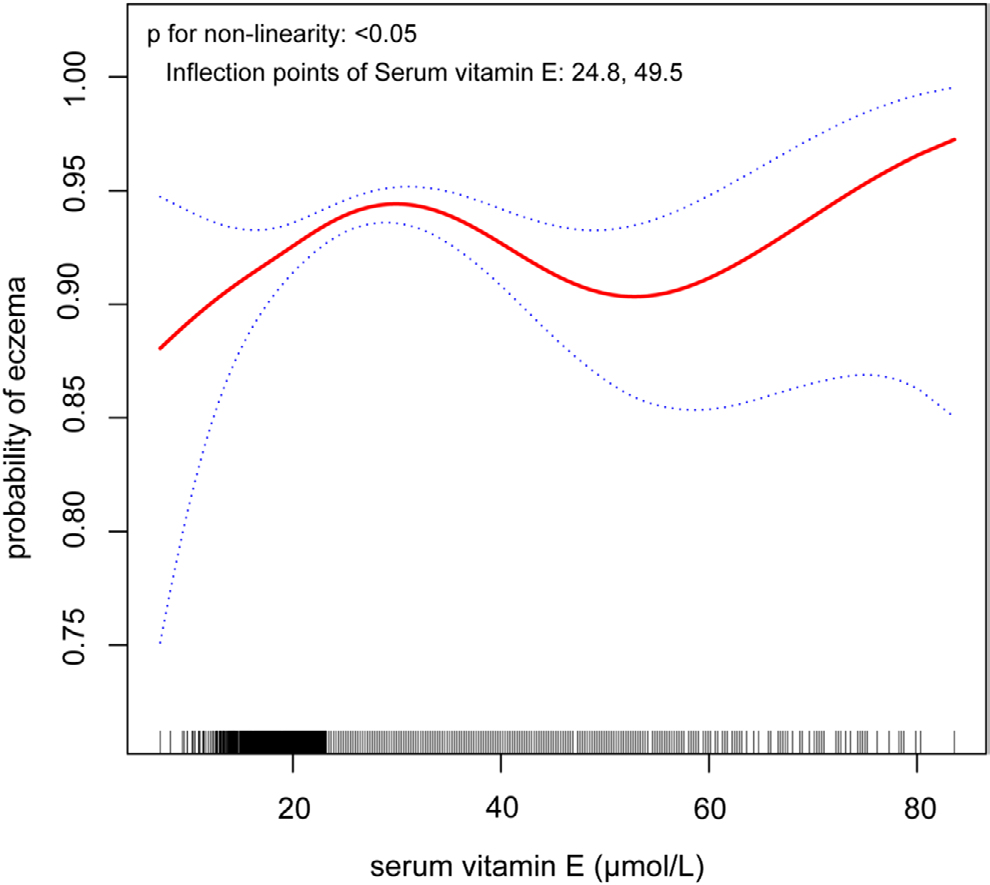
Non‐linear relationship between serum vitamin E and the incidence of eczema. After adjusting for age, gender, season, education level, PIR, BMI, smoking status, drinking alcohol status, depression, sleep, asthma, hay fever, and IgE, a non‐linear relationship was found.

A smoothing curve was constructed using a GAM to assess the potential non‐linear relationship between serum vitamin E and eczema. As shown in Figure 2, a non‐linear association was observed between serum vitamin E and the risk of eczema after adjusting for age, gender, season, education level, PIR, smoking status, drinking alcohol status, BMI, depressive symptoms, sleep duration, asthma, hay fever, and total IgE levels.

As shown in Table 4, to identify potential inflection points, a two‐piecewise linear regression model was applied. The analysis revealed two inflection points at 27.4 and 49.5 μmol/L (design‐adjusted Wald *F* test, *p* < 0.001). When serum vitamin E levels were below 27.4 μmol/L, a significant positive association was observed: each 1‐unit increase in serum vitamin E was associated with a 6.4% higher risk of eczema (OR = 1.064, 95% CI: 1.024–1.105; *p* = 0.001). Between 27.4 and 49.5 μmol/L, serum vitamin E was significantly inversely associated with eczema, with each 1‐unit increase corresponding to a 4% reduction in eczema risk (OR = 0.960, 95% CI: 0.934–0.987; *p* = 0.004). For serum vitamin E levels above 49.5 μmol/L, a positive association trend was observed, though it was not statistically significant (OR = 1.040, 95% CI: 0.976–1.108; *p* = 0.228).

**TABLE 4:** Threshold effect analysis of serum vitamin E on eczema using piecewise linear regression.

	Eczema (OR, 95% CI)	*p*
Fitting model by standard linear regression	1.000 (0.988, 1.013)	0.958
Fitting model by two‐piecewise linear regression		
Inflection points of serum vitamin E	27.4, 49.5	
< 27.4	1.064 (1.024, 1.105)	0.001
27.4–49.5	0.960 (0.934, 0.987)	0.004
> 49.5	1.040 (0.976, 1.108)	0.228
*p* for design‐adjusted Wald *F* test		< 0.001

*Note:* We adjusted for demographic variables (age, gender, season, education level, PRI), way of life variables (smoking status, drinking alcohol status, BMI), stress variables (depression, sleep), and clinical comorbidity variables (asthma, hay fever, IgE).

### 3.5 Sensitivity Analysis

Table S3 confirms complete data availability for both the exposure and outcome variables, while missing values were identified in several covariates, with the number of missing observations ranging from minimal (*n* = 3, 0.1% for smoking status) to substantial (*n* = 372, 8.4% for depression status).

As shown in Table S4, no significant differences (*p* > 0.05) were observed between the original data and the 5 sets of data generated by MI for all the variables. Figure S1 shows that the trends of the smoothing curves for the six data sets were basically the same. The sensitivity analyses described above allow us to determine that the analyses using the raw data are reliable.

## 4 Discussion

Our study reveals a significant nonlinear association between serum vitamin E levels (α‐tocopherol) and eczema prevalence in US adults, with a clear threshold effect. After adjusting for covariates, GAM analysis demonstrated that serum vitamin E concentrations between 27.4 and 49.5 μmol/L were negatively associated with eczema risk, while levels below 27.4 μmol/L showed a positive association. This biphasic association aligns with evidence from previous experimental studies demonstrating the biphasic modulatory effects of serum vitamin E levels (ElAttar and Lin 1992; Pal et al. 2003).

Our study deepens the understanding of the association between vitamin E and eczema. Previous findings on the relationship between vitamin E and eczema have been inconsistent. Oh et al. observed that children who consumed more dietary vitamin E were less likely to develop eczema, which led to higher serum α‐tocopherol levels (Oh et al. 2010). However, Miyake et al. reported that maternal vitamin E intake during pregnancy was not associated with the risk of eczema in the child (Miyake et al. 2010). The results of another study showed a significant negative association between serum α‐tocopherol levels and the prevalence of AD in Japanese students aged 10 to 13 years (Okuda et al. 2010). A significant negative connection was observed between serum α‐tocopherol and IgE levels in Korean infants and children with eczema, suggesting the importance of maintaining high serum α‐tocopherol in patients with eczema (Lee et al. 2012). However, the results of a cohort study of European children by Nwaru et al. showed no association between serum vitamin E concentrations in the first year of life and the risk of developing eczema over a 6‐year period (Nwaru et al. 2014).

Notably, although a previous NHANES‐based investigation indicated no significant linear association between serum vitamin E and eczema (Wei and Ji 2020), our study employed GAM analysis to reveal for the first time a nonlinear relationship between serum vitamin E levels and eczema in US adults, and identified a threshold effect that had been obscured in linear analyses.

Serum vitamin E between 27.4 and 49.5 μmol/L is negatively associated with eczema, suggesting that serum vitamin E may have a protective effect on eczema within this concentration range. As a potent lipid‐soluble antioxidant, α‐tocopherol neutralizes reactive oxygen species (ROS) that exacerbate epidermal barrier dysfunction (Gehin et al. 2006). Experimental studies demonstrate its capacity to suppress nuclear factor‐κB‐mediated inflammatory pathways (Hayashi et al. 2012), reducing key cytokines such as IL‐4 and IL‐13 (Hayashi et al. 2012; Mabalirajan et al. 2009), which drive eczema pathogenesis. Furthermore, α‐tocopherol enhances the expression of structural proteins critical for maintaining skin barrier integrity (Kato and Takahashi 2012). Several prospective observational studies have demonstrated that serum α‐tocopherol concentrations ≥ 30 μmol/L confer health benefits (Péter et al. 2015), and the present study further refines the definition of the “optimal range” for vitamin E in relation to eczema.

However, vitamin E levels < 27.4 μmol/L were positively associated with eczema, suggesting the existence of a critical threshold for antioxidant defense in skin homeostasis. Below this concentration, insufficient antioxidant defense may lead to oxidative stress accumulation and impaired barrier function. This helps to explain conflicting results from previous studies examining linear associations or different concentration ranges.

Our findings resonate with studies of other micronutrients in dermatology. For instance, Wei and Ji (2020) identified an inverted U‐shaped relationship between serum vitamin D and eczema risk, highlighting the importance of nutrient homeostasis in immune regulation. Similarly, vitamin E > 49.5 μmol/L in our study showed no significant association, potentially due to saturation of antioxidant pathways.

This research was performed utilizing the US CDC NHANES database, representing a nationally representative large‐scale population study that reflects the overall characteristics of the adult population in the United States (Wei et al. 2019). We adjusted for potential confounding covariates in the analysis, including, demographic, lifestyle, stress, and medical comorbidity variables. Using GAM, we were the first to characterize the nonlinear relationship between serum vitamin E (α‐tocopherol) levels and eczema prevalence, identifying the threshold saturation effect. Nonetheless, our study has several limitations. First, because of the cross‐sectional design of our study, the causal association between serum vitamin E and eczema could not be examined. Second, eczema status was a physician's diagnosis reported by the participants themselves, perhaps susceptible to recollection bias. This may have resulted in an underappraisal of eczema prevalence. However, the self‐report of eczema has been verified and displays a great association with clinical findings (Silverberg and Paller 2015). Third, the study focused solely on α‐tocopherol, while other vitamin E isoforms (e.g., γ‐tocopherol) may have distinct immunological effects. Although, as previously mentioned, α‐tocopherol is the main component and most biologically active form of vitamin E in the human body (Ağagündüz et al. 2023; Choi et al. 2020; Reboul et al. 2006). Future research should explore the roles of different vitamin E forms in eczema pathogenesis. Fourth, the results could not differentiate between eczema subtypes, including atopic and non‐atopic eczema, as NHANES data do not distinguish these classifications. Vitamin E may exhibit distinct biological interactions with different eczema subtypes. Despite these limitations, our study provides novel evidence of a nonlinear relationship between serum α‐tocopherol and eczema among adults in the US.

## 5 Conclusion

In this research, we found that serum vitamin E between 27.4 and 49.5 μmol/L was associated with a reduced risk of eczema, whereas levels < 27.4 μmol/L were associated with an increased risk of eczema. This result suggests that the effect of vitamin E on eczema may be bidirectional. In the future, we will conduct prospective and experimental research to confirm these findings and explore the underlying mechanisms.

## Author Contributions

**Tianhang Yu:** conceptualization (equal), data curation (equal), writing – review and editing (equal). **Peiyu Wang:** conceptualization (equal), formal analysis (equal), writing – review and editing (equal). **Suhua Wu:** conceptualization (equal), writing – original draft (equal). **Xueyun Cheng:** methodology (equal), writing – original draft (equal). **Linfeng Li:** supervision (equal), writing – review and editing (equal).

## Funding

The authors have nothing to report.

## Ethics Statement

The NHANES study protocol received approval from the NCHS Institutional Review Board, with written informed consent obtained from all participants.

## Conflicts of Interest

The authors declare no conflicts of interest.

## Supporting information


**Data S1:** fsn371265‐sup‐0001‐Supinfo01.docx.

## Data Availability

The datasets analyzed in this study are publicly available through the National Health and Nutrition Examination Survey (NHANES) program. All data can be accessed via the official NHANES repository: https://www.cdc.gov/nchs/nhanes/index.html.
