# Supplementary material for: Non‐Linear Association of Relationship Between Serum Vitamin E and Eczema in US Adults
Source: Food Sci Nutr. 2025 Nov 24;13(12):e71265. doi: 10.1002/fsn3.71265 (PMC12641440; doi:10.1002/fsn3.71265)
Supplement: Supplementary file 1 — Data S1: fsn371265‐sup‐0001‐Supinfo01.docx. [file FSN3-13-e71265-s001.docx]

**Supplemental Content**

**Supplemental Table 1:** Baseline characteristics of study participants according to serum vitamin E quartiles.

**Supplemental Table 2:** Relationship between serum vitamin E quartiles and eczema in different models.

**Supplemental Table 3:** Missing conditions for each variable.

**Supplemental Table 4:** Comparative analysis between the original data and the multiply imputed data regarding population characteristics.

**Supplemental Figure 1:** Comparative analysis of smoothed curves patterns between the original data (0) and multiply imputed data (1-5).

**Supplemental Table** **1:** Baseline characteristics of study participants according to serum vitamin E quartiles

| **Serum vitamin E (quartile)** | **Q1** **(＜21.20)** | **Q2 (21.22-26.24)** | **Q3 (26.47-33.67)** | **Q4 (＞33.90)** | ***P*-value** |
| --- | --- | --- | --- | --- | --- |
| Participants, n | 1107 | 1091 | 1118 | 1117 |  |
| Age, years | 38.11 (37.36 ,38.86) | 43.40 (42.23 ,44.56) | 48.64 (47.13 ,50.14) | 56.01 (54.05 ,57.97) | <0.0001 |
| Gender |  |  |  |  | 0.0028 |
| Male | 51.19 (47.39 ,54.98) | 48.88 (45.67 ,52.09) | 49.94 (45.88 ,54.00) | 42.61 (39.79 ,45.49) |  |
| Female | 48.81 (45.02 ,52.61) | 51.12 (47.91 ,54.33) | 50.06 (46.00 ,54.12) | 57.39 (54.51 ,60.21) |  |
| BMI, kg/m^2^ | 28.58 (27.88 ,29.27) | 28.44 (27.63 ,29.24) | 28.91 (28.28 ,29.54) | 28.41 (27.99 ,28.83) | 0.4704 |
| Total IgE, KU/L | 158.10 (136.99 ,179.21) | 131.93 (112.06 ,151.80) | 134.27 (116.70 ,151.84) | 99.50 (81.63 ,117.36) | 0.0019 |
| Race |  |  |  |  | <0.0001 |
| Mexican American | 9.29 (7.10 ,12.06) | 9.33 (6.60 ,13.04) | 7.05 (5.21 ,9.49) | 6.50 (4.46 ,9.36) |  |
| Other Hispanic | 3.46 (2.03 ,5.84) | 3.70 (1.90 ,7.09) | 3.92 (2.23 ,6.80) | 2.24 (1.22 ,4.10) |  |
| Non-Hispanic White | 60.41 (52.66 ,67.67) | 70.83 (64.36 ,76.55) | 76.20 (69.88 ,81.55) | 81.44 (75.25 ,86.36) |  |
| Non-Hispanic Black | 19.68 (14.66 ,25.89) | 12.05 (7.73 ,18.30) | 7.97 (5.34 ,11.71) | 5.27 (3.31 ,8.29) |  |
| Other Race | 7.16 (4.85 ,10.46) | 4.08 (2.97 ,5.60) | 4.86 (3.06 ,7.63) | 4.56 (3.07 ,6.71) |  |
| Education level |  |  |  |  | <0.0001 |
| Less than 9th grade | 5.99 (4.16 ,8.56) | 6.62 (4.88 ,8.93) | 6.29 (4.71 ,8.37) | 6.77 (4.94 ,9.23) |  |
| 9-11th grade | 16.26 (13.08 ,20.03) | 11.29 (8.40 ,15.02) | 10.40 (7.68 ,13.94) | 6.85 (5.31 ,8.81) |  |
| High school graduate | 26.88 (23.32 ,30.76) | 26.01 (23.34 ,28.87) | 21.64 (18.75 ,24.85) | 24.90 (20.35 ,30.08) |  |
| Some college or associate's degree | 35.38 (30.87 ,40.15) | 29.16 (24.24 ,34.61) | 32.26 (29.35 ,35.31) | 28.90 (24.88 ,33.28) |  |
| College graduate or higher | 15.50 (11.14 ,21.16) | 26.92 (21.14 ,33.61) | 29.41 (24.20 ,35.21) | 32.57 (26.93 ,38.77) |  |
| PIR | 2.66 (2.47 ,2.85) | 3.03 (2.85 ,3.21) | 3.33 (3.17 ,3.49) | 3.43 (3.27 ,3.60) | <0.0001 |
| Drinking alcohol status |  |  |  |  | 0.3717 |
| Yes | 72.25 (66.41 ,77.43) | 73.93 (68.71 ,78.56) | 76.38 (70.30 ,81.54) | 73.59 (69.53 ,77.28) |  |
| No | 27.75 (22.57 ,33.59) | 26.07 (21.44 ,31.29) | 23.62 (18.46 ,29.70) | 26.41 (22.72 ,30.47) |  |
| Smoking status |  |  |  |  | 0.0959 |
| Yes | 51.77 (47.06 ,56.44) | 48.61 (44.32 ,52.92) | 50.34 (46.12 ,54.56) | 45.00 (39.90 ,50.20) |  |
| No | 48.23 (43.56 ,52.94) | 51.39 (47.08 ,55.68) | 49.66 (45.44 ,53.88) | 55.00 (49.80 ,60.10) |  |
| Season |  |  |  |  | 0.0477 |
| Summer | 57.42 (40.98 ,72.36) | 63.97 (50.04 ,75.89) | 56.67 (43.33 ,69.11) | 57.02 (42.51 ,70.42) |  |
| Winter | 42.58 (27.64 ,59.02) | 36.03 (24.11 ,49.96) | 43.33 (30.89 ,56.67) | 42.98 (29.58 ,57.49) |  |
| Depression |  |  |  |  | 0.0123 |
| none | 77.48 (73.18 ,81.26) | 79.36 (75.36 ,82.85) | 82.23 (79.36 ,84.77) | 81.83 (78.48 ,84.77) |  |
| mild | 14.65 (11.26 ,18.84) | 14.72 (12.58 ,17.15) | 13.13 (10.99 ,15.61) | 14.72 (12.41 ,17.37) |  |
| moderate | 5.40 (3.92 ,7.42) | 4.17 (3.11 ,5.56) | 3.02 (2.06 ,4.41) | 2.13 (1.31 ,3.43) |  |
| severe | 2.47 (1.47 ,4.11) | 1.76 (0.88 ,3.50) | 1.62 (0.95 ,2.77) | 1.32 (0.68 ,2.53) |  |
| Sleep |  |  |  |  | <0.0001 |
| ＜6 h | 17.19 (15.39 ,19.16) | 15.62 (13.13 ,18.49) | 10.21 (7.69 ,13.44) | 10.49 (8.88 ,12.36) |  |
| 6‐7 h | 52.82 (49.11 ,56.49) | 51.33 (46.89 ,55.75) | 52.99 (49.41 ,56.54) | 53.54 (49.42 ,57.61) |  |
| ＞7 h | 29.99 (26.04 ,34.27) | 33.04 (29.29 ,37.03) | 36.80 (32.17 ,41.69) | 35.97 (31.80 ,40.36) |  |
| Eczema |  |  |  |  | 0.0114 |
| Yes | 9.60 (7.29 ,12.55) | 8.23 (6.79 ,9.94) | 5.08 (3.17 ,8.04) | 8.04 (5.60 ,11.43) |  |
| No | 90.40 (87.45 ,92.71) | 91.77 (90.06 ,93.21) | 94.92 (91.96 ,96.83) | 91.96 (88.57 ,94.40) |  |
| Asthma |  |  |  |  | 0.0014 |
| Yes | 16.97 (14.80 ,19.39) | 13.51 (11.20 ,16.21) | 15.17 (12.38 ,18.46) | 10.37 (8.10 ,13.19) |  |
| No | 83.03 (80.61 ,85.20) | 86.49 (83.79 ,88.80) | 84.83 (81.54 ,87.62) | 89.63 (86.81 ,91.90) |  |
| Hay fever |  |  |  |  | 0.0109 |
| Yes | 10.57 (8.09 ,13.69) | 11.48 (9.23 ,14.19) | 13.45 (10.51 ,17.06) | 16.26 (13.60 ,19.33) |  |
| No | 89.43 (86.31 ,91.91) | 88.52 (85.81 ,90.77) | 86.55 (82.94 ,89.49) | 83.74 (80.67 ,86.40) |  |

Results in table: For continuous variables: survey-weighted mean (95% CI), *P*-value was by survey-weighted linear regression. For categorical variables:

survey-weighted percentage (95% CI), *P*-value was by design-adjusted Rao–Scott chi-square test.

Q, quartile; BMI, body mass index; PIR, poverty income ratio, IgE, immunoglobin E, Serum vitamin E, α-tocopherol.

**Supplemental Table 2:** Relationship between serum vitamin E quartiles and eczema in different models

| **Serum vitamin E, μmol/L** | **Crude model (OR, 95% CI)** | ***p*** | **Model I (OR, 95% CI)** | ***p*** | **Model II (OR, 95% CI)** | ***p*** |
| --- | --- | --- | --- | --- | --- | --- |
| Serum vitamin E (quartile) |  |  |  |  |  |  |
| Q1 (＜21.20) | Ref |  | Ref |  | Ref |  |
| Q2 (21.22-26.24) | 1.267 (0.911, 1.762) | 0.159 | 1.257 (0.902, 1.751) | 0.177 | 1.203 (0.837, 1.728) | 0.318 |
| Q3 (26.47-33.67) | 1.727 (1.211, 2.462) | 0.003 | 1.674 (1.166, 2.404) | 0.005 | 1.858 (1.239, 2.787) | 0.003 |
| Q4 (33.90-83.59) | 1.122 (0.816, 1.543) | 0.479 | 1.089 (0.776, 1.528) | 0.621 | 1.049 (0.717, 1.534) | 0.807 |

Crude model: we did not adjust other covariates. Model I: we adjusted age, gender. Model II: we adjusted demographic variables (age, gender, season,

education level, PRI), way of life variables (smoking status, drinking alcohol status, BMI), stress variables (depression, sleep), and clinical comorbidity

variables (asthma, hay fever, IgE).

**Supplemental Table 3:** Missing situation for each variable

| **Variables** | **non-missing** | **missing** |
| --- | --- | --- |
| Gender | 4433(100%) | 0 (0%) |
| Age | 4433(100%) | 0 (0%) |
| Season | 4433(100%) | 0 (0%) |
| Race | 4433(100%) | 0 (0%) |
| Education level | 4428(99.9%) | 5(0.1%) |
| PIR | 4234(95.5%) | 199(4.5%) |
| BMI | 4368(98.5%) | 65(1.5%) |
| Drinking alcohol status | 4120(92.9%) | 313(7.1%) |
| Smoking status | 4430(99.9%) | 3(0.1%) |
| Eczema | 4427(99.9%) | 6(0.1%) |
| Hay fever | 4421(99.7%) | 12(0.3%) |
| IgE | 4426(99.8%) | 7(0.2%) |
| Eczema | 4433(100%) | 0 (0%) |
| Vitamin E | 4433(100%) | 0 (0%) |
| Depression | 4061(91.6%) | 372(8.4%) |
| Sleep | 4423(99.8%) | 10(2%) |

**Supplemental Table 4:** Comparative analysis between the original dataset and the multiply imputed datasets regarding population characteristics.

| **MI item** | **Pre-imputation** | **Pro-imputation 1** | **Pro-imputation 2** | **Pro-imputation 3** | **Pro-imputation 4** | **Pro-imputation 5** | ***p*** |
| --- | --- | --- | --- | --- | --- | --- | --- |
| Participants | 4433 | 4433 | 4433 | 4433 | 4433 | 4433 |  |
| Vitamin E, μmol/L | 28.94 (11.27) | 28.94 (11.27) | 28.94 (11.27) | 28.94 (11.27) | 28.94 (11.27) | 28.94 (11.27) | 1.000 |
| Age | 48.30 (18.85) | 48.30 (18.85) | 48.30 (18.85) | 48.30 (18.85) | 48.30 (18.85) | 48.30 (18.85) | 1.000 |
| PIR | 2.42 (1.25-4.24) | 2.42 (1.25-4.20) | 2.41 (1.24-4.18) | 2.41 (1.24-4.18) | 2.41 (1.25-4.18) | 2.41 (1.24-4.18) | 0.995* |
| BMI | 28.81 (6.76) | 28.81 (6.75) | 28.79 (6.76) | 28.79 (6.76) | 28.79 (6.77) | 28.79 (6.75) | 1.000 |
| IgE | 45.90 (17.60-129.00) | 45.90 (17.60-129.00) | 45.90 (17.60-129.00) | 45.90 (17.60-129.00) | 45.90 (17.60-129.00) | 45.90 (17.60-129.00) | 1.000* |
| Hay fever |  |  |  |  |  |  | 1.000 |
| Yes | 460 (10.40%) | 462 (10.42%) | 462 (10.42%) | 463 (10.44%) | 463 (10.44%) | 463 (10.44%) |  |
| No | 3961 (89.60%) | 3971 (89.58%) | 3971 (89.58%) | 3970 (89.56%) | 3970 (89.56%) | 3970 (89.56%) |  |
| Eczema |  |  |  |  |  |  | 1.000 |
| Yes | 284 (6.41%) | 284 (6.41%) | 284 (6.41%) | 284 (6.41%) | 284 (6.41%) | 284 (6.41%) |  |
| No | 4149 (93.59%) | 4149 (93.59%) | 4149 (93.59%) | 4149 (93.59%) | 4149 (93.59%) | 4149 (93.59%) |  |
| Season |  |  |  |  |  |  | 1.000 |
| Summer | 2414 (54.46%) | 2414 (54.46%) | 2414 (54.46%) | 2414 (54.46%) | 2414 (54.46%) | 2414 (54.46%) |  |
| Winter | 2019 (45.54%) | 2019 (45.54%) | 2019 (45.54%) | 2019 (45.54%) | 2019 (45.54%) | 2019 (45.54%) |  |
| Gender |  |  |  |  |  |  | 1.000 |
| Male | 2138 (48.23%) | 2138 (48.23%) | 2138 (48.23%) | 2138 (48.23%) | 2138 (48.23%) | 2138 (48.23%) |  |
| Female | 2295 (51.77%) | 2295 (51.77%) | 2295 (51.77%) | 2295 (51.77%) | 2295 (51.77%) | 2295 (51.77%) |  |
| Race |  |  |  |  |  |  | 1.000 |
| Mexican American | 902 (20.35%) | 902 (20.35%) | 902 (20.35%) | 902 (20.35%) | 902 (20.35%) | 902 (20.35%) |  |
| Other Hispanic | 136 (3.07%) | 136 (3.07%) | 136 (3.07%) | 136 (3.07%) | 136 (3.07%) | 136 (3.07%) |  |
| Non-Hispanic White | 2240 (50.53%) | 2240 (50.53%) | 2240 (50.53%) | 2240 (50.53%) | 2240 (50.53%) | 2240 (50.53%) |  |
| Non-Hispanic Black | 983 (22.17%) | 983 (22.17%) | 983 (22.17%) | 983 (22.17%) | 983 (22.17%) | 983 (22.17%) |  |
| Other Race | 172 (3.88%) | 172 (3.88%) | 172 (3.88%) | 172 (3.88%) | 172 (3.88%) | 172 (3.88%) |  |
| Education level |  |  |  |  |  |  | 1.000 |
| Less than 9th grade | 545 (12.31%) | 547 (12.34%) | 548 (12.36%) | 549 (12.38%) | 547 (12.34%) | 548 (12.36%) |  |
| 9-11th grade | 682 (15.40%) | 684 (15.43%) | 683 (15.41%) | 683 (15.41%) | 682 (15.38%) | 682 (15.38%) |  |
| High school graduate | 1046 (23.62%) | 1047 (23.62%) | 1047 (23.62%) | 1046 (23.60%) | 1049 (23.66%) | 1047 (23.62%) |  |
| Some college or associate's degree | 1268 (28.64%) | 1268 (28.60%) | 1268 (28.60%) | 1268 (28.60%) | 1268 (28.60%) | 1269 (28.63%) |  |
| College graduate or above | 887 (20.03%) | 887 (20.01%) | 887 (20.01%) | 887 (20.01%) | 887 (20.01%) | 887 (20.01%) |  |
| Drinking alcohol status |  |  |  |  |  |  | 0.613 |
| Yes | 2837 (68.86%) | 3016 (68.04%) | 2997 (67.61%) | 2985 (67.34%) | 2986 (67.36%) | 2984 (67.31%) |  |
| No | 1283 (31.14%) | 1417 (31.96%) | 1436 (32.39%) | 1448 (32.66%) | 1447 (32.64%) | 1449 (32.69%) |  |
| Smoking status |  |  |  |  |  |  | 1.000 |
| Yes | 2102 (47.45%) | 2103 (47.44%) | 2103 (47.44%) | 2103 (47.44%) | 2102 (47.42%) | 2103 (47.44%) |  |
| No | 2328 (52.55%) | 2330 (52.56%) | 2330 (52.56%) | 2330 (52.56%) | 2331 (52.58%) | 2330 (52.56%) |  |
| Asthma |  |  |  |  |  |  | 1.000 |
| Yes | 571 (12.90%) | 571 (12.88%) | 571 (12.88%) | 572 (12.90%) | 571 (12.88%) | 571 (12.88%) |  |
| No | 3856 (87.10%) | 3862 (87.12%) | 3862 (87.12%) | 3861 (87.10%) | 3862 (87.12%) | 3862 (87.12%) |  |
| Depression |  |  |  |  |  |  | 1.000 |
| none | 3227 (79.46%) | 3513 (79.25%) | 3528 (79.58%) | 3537 (79.79%) | 3530 (79.63%) | 3536 (79.77%) |  |
| mild | 581 (14.31%) | 638 (14.39%) | 626 (14.12%) | 626 (14.12%) | 628 (14.17%) | 626 (14.12%) |  |
| moderate | 170 (4.19%) | 185 (4.17%) | 190 (4.29%) | 186 (4.20%) | 187 (4.22%) | 184 (4.15%) |  |
| severe | 83 (2.04%) | 97 (2.19%) | 89 (2.01%) | 84 (1.89%) | 88 (1.99%) | 87 (1.96%) |  |
| Sleep |  |  |  |  |  |  | 1.000 |
| 6 h | 667 (15.08%) | 669 (15.09%) | 668 (15.07%) | 668 (15.07%) | 669 (15.09%) | 669 (15.09%) |  |
| 6‐7 h | 2175 (49.17%) | 2180 (49.18%) | 2180 (49.18%) | 2180 (49.18%) | 2181 (49.20%) | 2178 (49.13%) |  |
| 7+ h | 1581 (35.74%) | 1584 (35.73%) | 1585 (35.75%) | 1585 (35.75%) | 1583 (35.71%) | 1586 (35.78%) |  |

Mean (SD) Median (Q1-Q3) / N (%)

p-value*: For continuous variables, derived using the Kruskal-Wallis rank-sum test; for categorical variables with expected counts <10, derived using Fisher's exact probability test

*
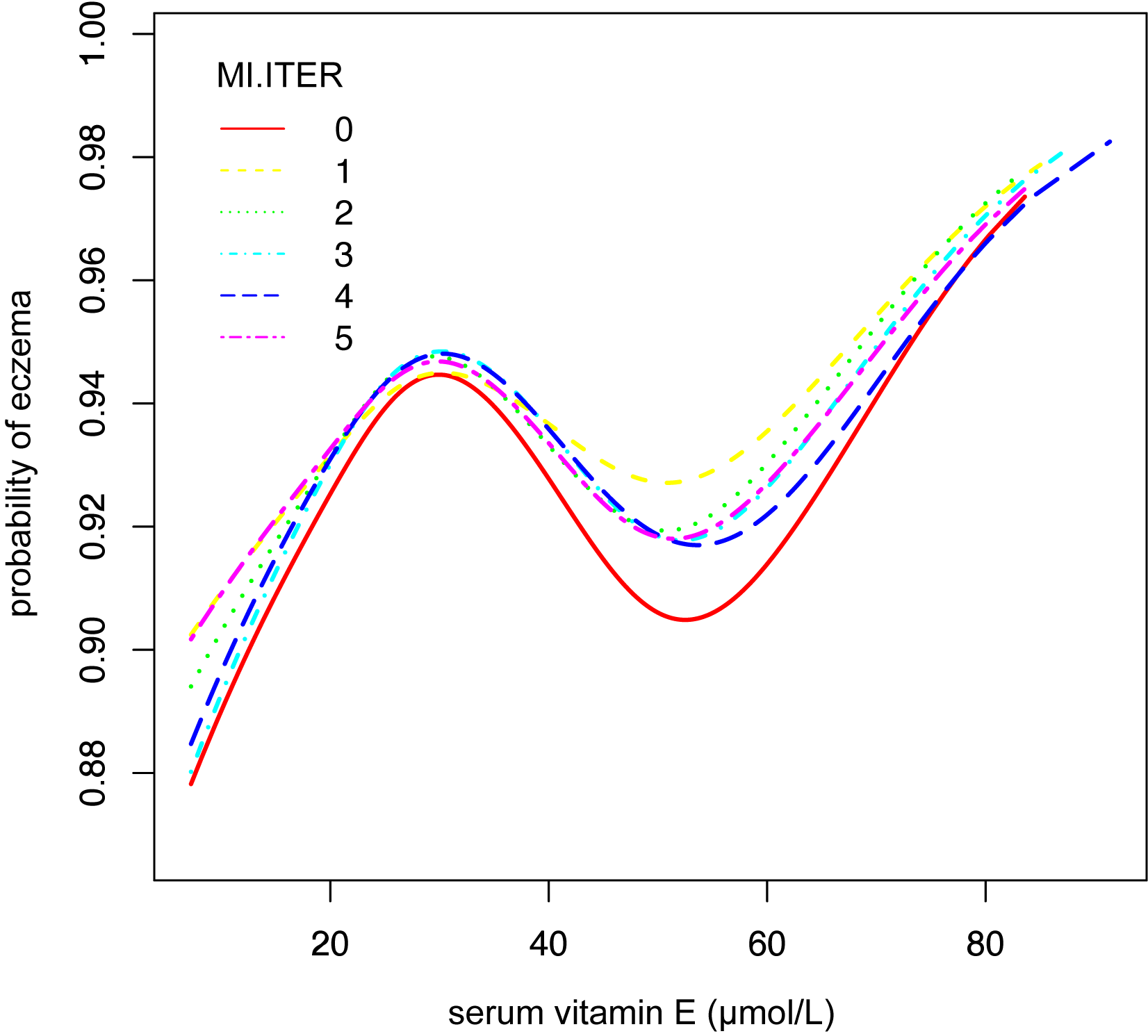
*

**Supplemental Figure 1:** Comparative analysis of smoothed curves patterns between the original data (0) and multiply imputed data (1-5).
